# Selective Augmentation of Stem Cell Populations in Structural Fat Grafts for Maxillofacial Surgery

**DOI:** 10.1371/journal.pone.0110796

**Published:** 2014-11-06

**Authors:** Luigi Clauser, Letizia Ferroni, Chiara Gardin, Riccardo Tieghi, Manlio Galiè, Giovanni Elia, Adriano Piattelli, Paolo Pinton, Eriberto Bressan, Barbara Zavan

**Affiliations:** 1 Unit of Cranio Maxillo Facial Surgery, Center for Craniofacial Deformities and Orbital Surgery – Reference Center for Rare Disease, Sant'Anna Hospital and University, Cona, Ferrara, Italy; 2 Department of Biomedical Sciences, University of Padova, Padova, Italy; 3 Department of Medical, Oral, and Biotechnological Sciences, University of Chieti-Pescara, Chieti, Italy; 4 Department of Morphology, Surgery and Experimental Medicine, Section of Pathology Oncology and Experimental Biology, University of Ferrara, Ferrara, Italy; 5 Department of Neurosciences, University of Padova, Padova, Italy; University of Sheffield, United Kingdom

## Abstract

Structural fat grafting utilizes the centrifugation of liposuction aspirates to create a graded density of adipose tissue. This study was performed to qualitatively investigate the effects of centrifugation on stem cells present in adipose tissue. Liposuction aspirates were obtained from healthy donors and either not centrifuged or centrifuged at 1,800 rpm for 3 minutes. The obtained fat volumes were divided into three layers and then analyzed. The results demonstrate that centrifugation induces a different distribution of stem cells in the three layers. The high-density layer displays the highest expression of mesenchymal stem cell and endothelial markers. The low-density layer exhibits an enrichment of multipotent stem cells. We conclude that appropriate centrifugation concentrates stem cells. This finding may influence the clinical practice of liposuction aspirate centrifugation and enhance graft uptake.

## Introduction

Over the past two decades, the treatment of soft tissue loss with structural fat grafting (SFG) has yielded encouraging clinical results in terms of the texture, softness, and quality of skin patterns [1]–[3]. Autologous fat tissue transfer is thought to provide an ideal filler for soft tissue augmentation because fat tissue is biocompatible, versatile, natural looking, readily available, abundant, and inexpensive and because fat tissue can be harvested easily and repeatedly with minimal trauma to the donor sites. Implanted fat has the potential to be permanent; however, this fat can be removed if required [4]–[6]. Fat grafting has become increasingly popular because of the ability to remove fat from sites of excess fat and then implant the fat into sites of fat deficiency, allowing the body to be sculpted. This procedure was proposed by Coleman and was first applied for facial remodeling and for breast reconstruction and augmentation [7]–[10]. This procedure is typically considered safe and involves autologous tissue injection at a defective site directly after harvesting without ex vivo expansion [11]–[15]. However, fat grafting is not without disadvantages. Infection and damage to local nerves, muscles, glands, and blood vessels during harvest are always possible [7], [16]–[18]. Several studies have also reported disappointing short-term survival rates for implanted fat or excessive growth of transplanted fat. Irregular reabsorption remains a problem when using autologous, non-vascularized free-fat grafts to correct soft tissue defects [19]–[23]. Growth factors, beta-blockers, insulin, growth media, and hyperbaric oxygen have been used to prevent irregular resorption [24]. Although satisfactory results can be achieved during the early postoperative period, long-term results may be disappointing for both the patient and surgeon [25]–[27]. The most common difficulty is the estimated reabsorption rate. In light of such considerations, despite the widespread clinical use of SFG, little is known regarding the cell quality (the most important factor in fat grafting) when the tissue is injected into the patient. Therefore, the aim of the present study was to analyze the stemness properties of cells within fat that was aspirated and centrifuged according to Coleman's technique. Specifically, we performed molecular and cellular analyses of the stemness properties of cells distributed in the fat following the centrifugation required by Coleman's procedure.

## Materials and Methods

### Ethics statement

The Ethical Committee of Ferrara Hospital, Ferrera, Italy approved the research protocol. Written informed consent was obtained from all patients, in accordance with the Helsinki Declaration, before their inclusion in this study.

### Coleman's procedure

Using Coleman's procedure, fat was collected at the Cranio Maxillo Facial Surgery Unit at St. Anna Hospital, Ferrera, Italy and at the University of Ferrara, Ferrera, Italy from 30 healthy patients (age: 21–36 years old; BMI: 30–38; 25 females, 5 males) under general anesthesia; the patients were undergoing cosmetic surgery procedures. The fat donor sites were the abdominal area, love handles, inner sides of the thigh and knees, and flanks. The fat was collected using a cannula (Byron Medical (a division of Mentor Corporation), Tucson, AZ, USA) connected to a 10 ml Luer-Lok syringe (BD Syringe Luer-Lok tip; Becton Dickinson, Franklin Lakes, NJ, USA). The samples were sealed and centrifuged at 1800 rpm (rotor size: 16 cm; g force: 580) for 3 min. After centrifugation, the samples were separated into three layers: an upper yellow layer of oil derived from destroyed fat fragments, a middle layer composed of the adipose tissue graft, and a bottom layer of blood. The upper layer was discarded by ejection after rotating the syringe. Codman neuropads (Codman Neuro Sciences, Sarl, Switzerland) were used to wick the residual oil component. The lower layer was discarded by opening the plug from the Luer-Lok connection. The middle layer of fat was divided into three parts:

- the first 3 ml is referred to as the low-density layer (LDL),
- the central 3 ml is the middle-density layer (MDL), and
- the final 3 ml is the high-density layer (HDL), which is in the lower part of the syringe near the needle (obtained after the removal of red cells).

### DNA content

The DNA content was determined using a DNeasy kit (Qiagen, Hilden, Germany) to isolate the total DNA from cell cultures following the manufacturer's protocol for tissue isolation and an overnight incubation period with proteinase K (Qiagen). The DNA concentration was determined by measuring the absorbance at 260 nm using a spectrophotometer. Then, the cell number was determined using a standard curve (micrograms of DNA vs. cell number) generated by DNA extraction from counted cells. The standard curve was linear over the tested range of 5–80 µg DNA (r = 0.99)

### Real-time PCR

Total RNA was extracted using an RNeasy Lipid Tissue kit (Qiagen), including DNase digestion with the RNase-Free DNase Set (Qiagen), from the adipose tissue of the three different layers of the centrifuged lipoaspirate and from non-centrifuged lipoaspirate samples. In total, 800 ng of RNA was reverse-transcribed using an RT^2^ First Strand kit (Qiagen). Real-time PCR was performed according to the user's manual for the Human Mesenchymal Stem Cell RT2 Profiler PCR Array (SABiosciences, Frederick, MD, USA) and using RT^2^ SYBR Green ROX FAST Master Mix (Qiagen). Thermal cycling and fluorescence detection were performed using a Rotor-Gene Q 100 (Qiagen). The data were analyzed using Excel-based PCR Array Data Analysis Templates (SABiosciences). The results are reported as ratios with respect to the mRNA expression levels of non-centrifuged fat.

### Oil red O (ORO) staining

LDL-, MDL-, and HDL-derived cells were fixed in 4% formalin and stained with 0.3% ORO (Sigma-Aldrich, St. Louis, MO, USA) in 60% isopropanol. After washing with 60% isopropanol, the cells were acquired, and the dye was extracted with 100% isopropanol. The optical density (OD) of each solution was measured at 520 nm using a Victor 3 spectrophotometer (Perkin Elmer, Waltham, MA, USA).

### Immunocytochemical staining

The cells were layered and fixed on coverslips and then incubated in 2% bovine serum albumin (BSA, Sigma-Aldrich) solution in phosphate-buffered saline (PBS) for 30 minutes at room temperature. Then, the sections were incubated with the primary antibodies in 2% BSA solution in a humidified chamber overnight at 4°C. The following primary antibodies were used: rabbit polyclonal anti-human osteonectin antibody (1∶1000, AB1858, Millipore Corporation, MA, USA), chicken polyclonal anti-human Tubulin β3 antibody (1∶1000, AB9354, Millipore Corporation), rabbit polyclonal anti-human GFAP antibody (1∶100, AB5804, Millipore Corporation), rabbit polyclonal anti-human von Willebrand Factor antibody (1∶100, A0082, Dako, Milan, Italy), mouse monoclonal anti-human CD31 antibody (10 µg/ml, ab24590, Abcam, Cambridge, UK), rabbit polyclonal anti-human CD73 antibody (1∶100, ab71822, Abcam), rabbit monoclonal anti-human CD90 antibody (1∶100, ab92574, Abcam), and rabbit polyclonal anti-human CD105 antibody (1∶100, sc-20632, Santa Cruz Biotechnology, Inc., Santa Cruz, CA, USA). Immunofluorescence staining was performed using the secondary antibodies DyLight 549-labeled anti-rabbit IgG (H+L) (KPL, Gaithersburg, MD, USA), DyLight 488-labeled anti-mouse IgG (KPL), and DyLight 549-labeled anti-chicken IgG (H+L) (KPL), which were diluted 1∶1000 in 2% BSA for 1 hour at room temperature. Nuclear staining was performed with 2 µg/ml Hoechst H33342 (Sigma-Aldrich) solution for 2 minutes.

### Cell cultures

The lipoaspirate from the HDL was washed with PBS and digested using a solution of 0.075% collagenase from *Clostridium histolyticum* type II (Sigma-Aldrich, St. Louis, MO, USA) in Hank's balanced salt solution (HBSS, Lonza Srl, Milano, Italy) for 3 h at room temperature under slow agitation. At the end of the digestion, the collagenase activity was blocked with an equal volume of complete (cDMEM), which consisted of Dulbecco's modified Eagle's medium (DMEM, Lonza, Italy) supplemented with 10% fetal bovine serum (FBS, Bidachem SpA, Milano, Italy) and 1% penicillin/streptomycin (P/S, EuroClone). After centrifugation for 4 min at 1200 rpm, the pellet was washed in PBS and filtered using a 70-µM cell strainer (BD Biosciences, Mississauga, Ontario, Canada). The cell suspension was resuspended in cDMEM, transferred to a 25-cm^2^ tissue culture flask, and then incubated at 37°C and 5% CO_2_.

Subsequently, cells were seeded into seven 35-mm Petri dishes (50,000 cells/dish) and on seven scaffolds (see *3D scaffolds* section, 500,000/cm^2^) and then cultured in seven differentiation media:

1. Standard medium (Nonhematopoietic (NH) stem cell medium, Miltenyi Biotec, Bergish Gladbach, Germany);
2. Adipogenic differentiation medium (StemMACS AdipoDiff Media, Miltenyi Biotec);
3. Endothelial differentiation medium: DMEM containing 10% FBS, 10 ng/ml ECGF (Calbiochem, San Diego, CA, USA), 10 ng/ml bFGF (Prospec, East Brunswick, NJ, USA), 100 ng/ml porcine heparin (Seromed, Berlin, Germany);
4. Chondrogenic differentiation medium (StemMACS ChondroDiff Media, Miltenyi Biotec);
5. Osteogenic differentiation medium (StemMACS OsteoDiff Media, Miltenyi Biotec);
6. Neuronal differentiation medium: NeuroBasal medium (Gibco by Life Technologies, Paisley, UK) containing 1% FBS, 2% B27 (Gibco), NGF (Prospec), 100 ng/ml BDNF (Prospec), and 20 ng/ml NT3 (Prospec);
7. Glial differentiation medium: NeuroBasal medium containing 1% FBS, 1% N2 supplement (Gibco), 4 µM forskolin (Merck Millipore, Darmstadt, Germany), and 10 ng/ml heregulin β (Prospec).

The cells were cultured as a monolayer or on 3D scaffolds in standard, endothelial, adipogenic, and chondrogenic media for up to 21 days and in osteogenic, neurogenic and glial media for up to 28 days. The media were changed every 3 days.

### 3D scaffolds

Scaffold for osteogenic, endothelial, neuronal, and glial commitment: 1×1 cm non-woven meshes made of a linear derivative of hyaluronic acid (HYA) (HYAFF 11, Fidia Advanced Biopolymers, Abano Terme, Padova, Italy).

Scaffold for adipogenic commitment: cylindrical sponges made of HYA (HYAFF 11).

Scaffold for chondrogenic commitment: 1×1 cm bovine-derived pericardium membrane (Chondro-Gide, Geistlich Pharma AG, Wolhusen, Switzerland).

### Scanning electron microscopy (SEM)

Samples were fixed with 2.5% glutaraldehyde (glutaraldehyde solution Grade I, 25% in H_2_O; G5882, Sigma-Aldrich) in 0.1 M cacodylate (sodium cacodylate trihydrate; C0250, Sigma-Aldrich) buffer for 1 h before critical-point drying, followed by gold-palladium coating. All micrographs were obtained using a JEOL 6360LV SEM microscope (JEOL, Tokyo, Japan). The SEM analysis was performed at the Interdepartmental Service Center (CUGAS, University of Padova, Padova, Italy).

### Statistical analysis

One-way analysis of variance (ANOVA) was used for the data analyses. A repeated-measures ANOVA with a post-hoc analysis using Bonferroni's correction for multiple comparisons was performed, and t-tests were used to determine significant differences (p<0.05). Repeatability was calculated as the standard deviation of the difference between measurements. All testing was performed using the SPSS 16.0 software (SPSS Inc., Chicago, IL, USA) (licensed by the University of Padova).

## Results

**Figure 1:**
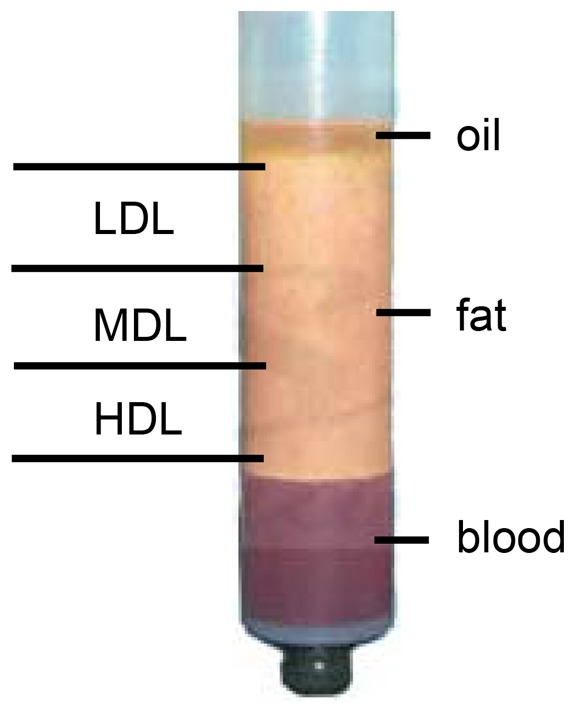
Coleman's procedure. After centrifugation for 3 min at 1800 rpm, the fat sample is separated into three layers: an upper yellow layer of oil, a middle layer of adipose tissue, and a bottom layer of blood. The top and bottom layers are discarded. The middle layer is divided into three distinct layers: a low-density layer (LDL), a middle-density layer (MDL), and a high-density layer (HDL).

Using Coleman's procedure, the abdominal fat of healthy patients was aspirated and centrifuged, thus producing three layers: an upper yellow layer of oil, a middle layer of adipose tissue, and a bottom layer of blood. The top and bottom layers were discarded. The middle layer was divided into three distinct parts: a low-density layer (LDL), a middle-density layer (MDL), and a high-density layer (HDL) (Figure 1A). The cell quantity of each layer was determined by measuring the DNA content (Figure 1B). The quantity of cells was similar for each subsequently analyzed layer.

Molecular, morphological and cellular analyses were performed on these layers.

**Figure 2:**
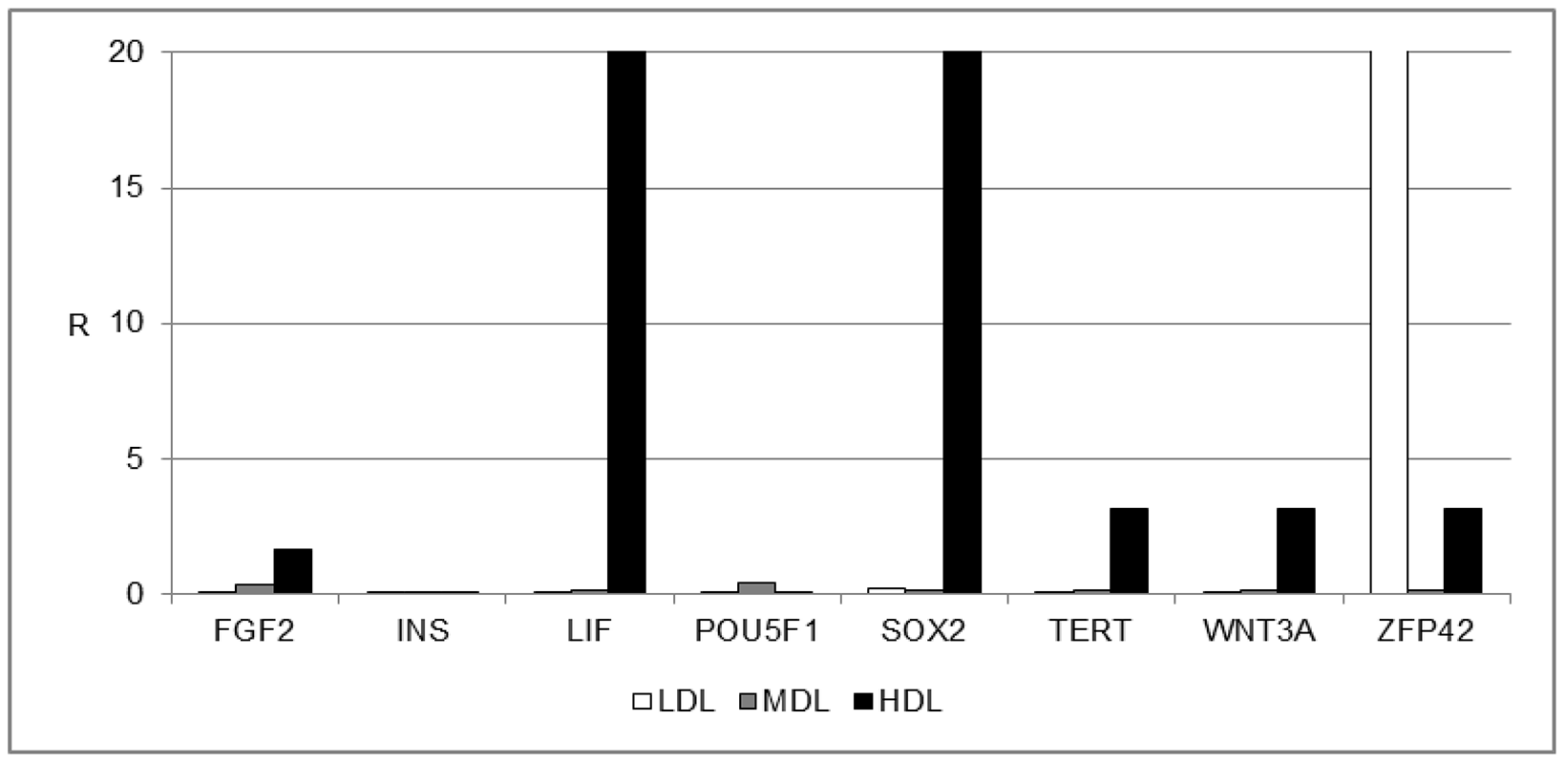
Gene expression profile of stemness markers. Gene expression profile of stemness markers in the LDL (white bars), MDL (grey bars), and HDL (black bars). The results are reported as ratios (R) with respect to the mRNA expression levels of non-centrifuged fat (not shown).

To characterize the stemness properties of the cells within the fat, the mRNA from each layer was extracted and analyzed by real-time PCR. The analyzed genes are listed in Table 1. Figure 2 shows the gene expression of stemness markers in the LDL, MDL, and HDL. Non-centrifuged fat was used as a control. Significant mRNA expression was detected for LIF, SOX2, TERT, WNT3A, and ZFP42. The HDL exhibited higher levels of LIF and SOX2 gene expression compared with those expression levels in the MDL and in the LDL, whereas the LDL displayed higher expression of the ZFP42 gene, which is a marker of pluripotent stem cells [28].

**Table 1:** List of genes analyzed by real-time PCR.

Gene symbol	Description
**ABCB1**	ATP-binding cassette, sub-family B member 1
**ALCAM**	Activated leukocyte cell adhesion molecule
**ANPEP**	Alanyl (membrane) aminopeptidase
**ANXA5**	Annexin A5
**BDNF**	Brain-derived neurotrophic factor
**BGLAP**	Bone gamma-carboxyglutamate (gla) protein
**BMP2**	Bone morphogenetic protein 2
**BMP4**	Bone morphogenetic protein 4
**BMP6**	Bone morphogenetic protein 6
**BMP7**	Bone morphogenetic protein 7
**CASP3**	Caspase 3, apoptosis-related cysteine peptidase
**CD44**	Cluster of differentiation 44 molecule
**COL1A1**	Collagen, type I, alpha 1
**CSF2**	Colony stimulating factor 2
**CSF3**	Colony stimulating factor 3
**CTNNB1**	Catenin (cadherin-associated protein), beta 1
**EGF**	Epidermal growth factor
**ENG**	Endoglin (CD105)
**ERBB2**	v-erb-b2 avian erythroblastic leukemia viral oncogene homolog 2
**FGF10**	Fibroblast growth factor 10
**FGF2**	Fibroblast growth factor 2
**FUT1**	Fucosyltransferase 1
**FUT4**	Fucosyltransferase 4
**FZD9**	Frizzled family receptor 9
**GDF15**	Growth differentiation factor 15
**GDF5**	Growth differentiation factor 5
**GDF7**	Growth differentiation factor 7
**GTF3A**	General transcription factor IIIA
**HAT1**	Histone acetyltransferase 1
**HDAC1**	Histone deacetylase 1
**HGF**	Hepatocyte growth factor
**HNF1A**	HNF1 homeobox A
**ICAM1**	Intercellular adhesion molecule 1
**IFNG**	Interferon, gamma
**IGF1**	Insulin-like growth factor 1
**IL10**	Interleukin 10
**IL1B**	Interleukin 1, beta
**IL6**	Interleukin 6
**INS**	Insulin
**ITGA6**	Integrin, alpha 6
**ITGAV**	Integrin, alpha V
**ITGAX**	Integrin, alpha X
**ITGB1**	Integrin, beta 1
**KDR**	Kinase insert domain receptor
**KITLG**	KIT ligand
**LIF**	Leukemia inhibitory factor
**MCAM**	Melanoma cell adhesion molecule
**MITF**	Microphthalmia-associated transcription factor
**MMP2**	Matrix metallopeptidase 2
**NES**	Nestin
**NGFR**	Nerve growth factor receptor
**NT5E**	5′-nucleotidase (CD73)
**NUDT6**	Nudix (nucleoside diphosphate linked moiety X)-type motif 6
**KAT2B**	K(lysine) acetyltransferase 2B
**PDGFRB**	Platelet-derived growth factor receptor, beta polypeptide
**PIGS**	Phosphatidylinositol glycan anchor biosynthesis, class S
**POU5F1**	POU class 5 homeobox 1
**PROM1**	Prominin 1
**PTK2**	PTK2 protein tyrosine kinase 2
**PTPRC**	Protein tyrosine phosphatase, receptor type, C
**RUNX2**	Runt-related transcription factor 2
**SLC17A5**	Solute carrier family 17, member 5
**SMAD4**	SMAD family member 4
**SMURF2**	SMAD specific E3 ubiquitin protein ligase 2
**SOX2**	SRY (sex determining region Y)-box 2
**SOX9**	SRY (sex determining region Y)-box 9
**TBX5**	T-box 5
**TERT**	Telomerase reverse transcriptase
**TGFB1**	Transforming growth factor, beta 1
**TGFB3**	Transforming growth factor, beta 3
**THY1**	Thy-1 cell surface antigen
**TNF**	Tumor necrosis factor
**VCAM1**	Vascular cell adhesion molecule 1
**VEGFA**	Vascular endothelial growth factor A
**VIM**	Vimentin
**VWF**	Von Willebrand factor
**WNT3A**	Wingless-type MMTV integration site family, member 3A
**ZFP42**	Zinc finger protein 42 homolog

**Figure 3:**
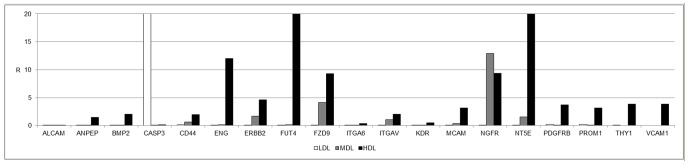
Gene expression profile of mesenchymal stem cell-specific markers. Gene expression profile of mesenchymal stem cell-specific markers in the LDL (white bars), MDL (grey bars), and HDL (black bars). The results are reported as ratios (R) with respect to the mRNA expression levels of non-centrifuged fat (not shown).

The characteristics of cells from the different layers were analyzed in greater detail. Figure 3 shows the expression of important markers for several cell processes, such as vascularization, which is mediated by stem cells (ENG) and by mesenchymal stem cells (CD44, FZD9, and NT5E), and migration and functional properties related to stemness, such as FUT4. The expression levels of all these markers were higher in the HDL than in the MDL or the LDL.

**Figure 4:**
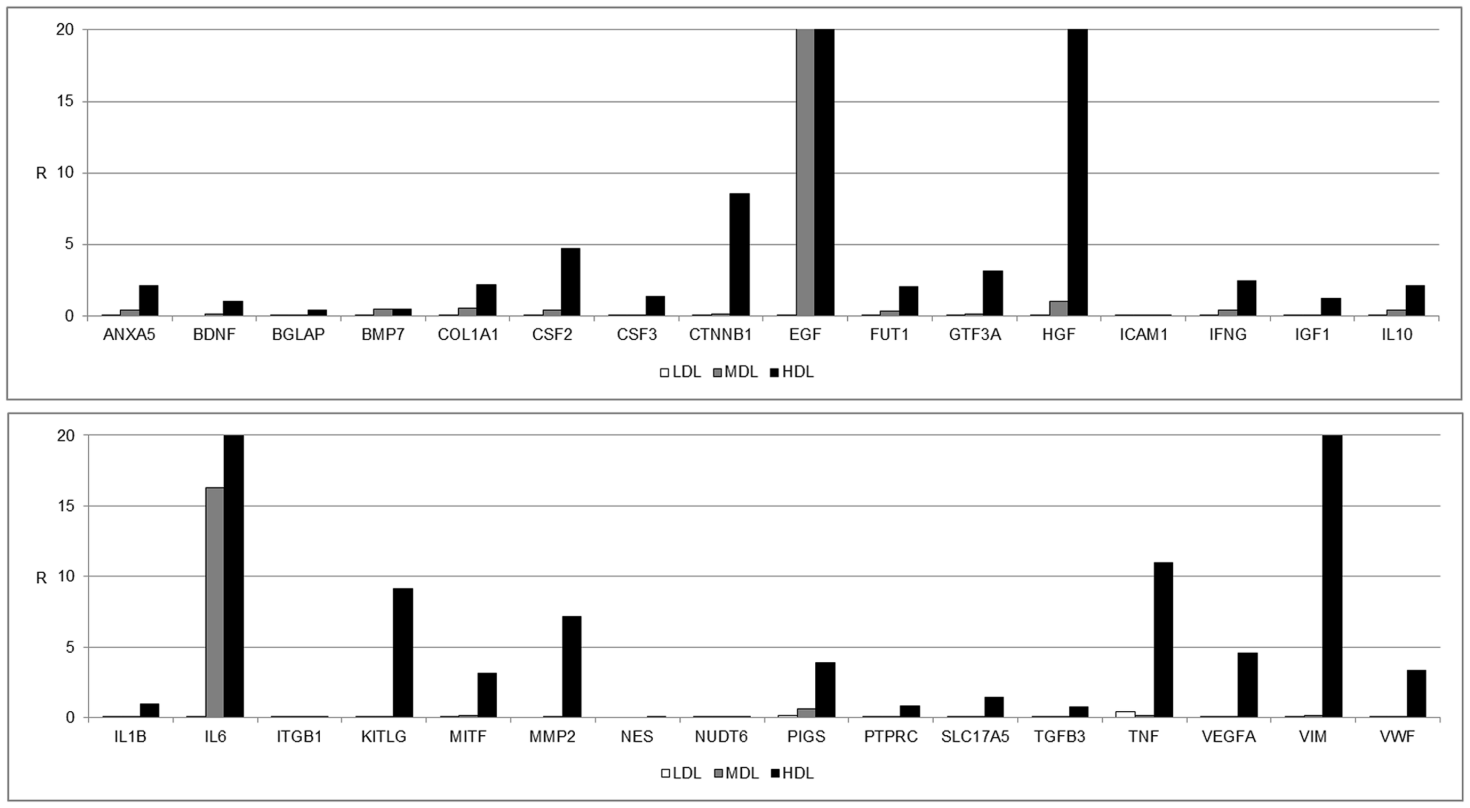
Gene expression profile of genes associated with mesenchymal stem cell markers. Gene expression profile of genes associated with mesenchymal stem cell markers in the LDL (white bars), MDL (grey bars), and HDL (black bars). The results are reported as ratios (R) with respect to the mRNA expression levels of non-centrifuged fat (not shown).

**Figure 5:**
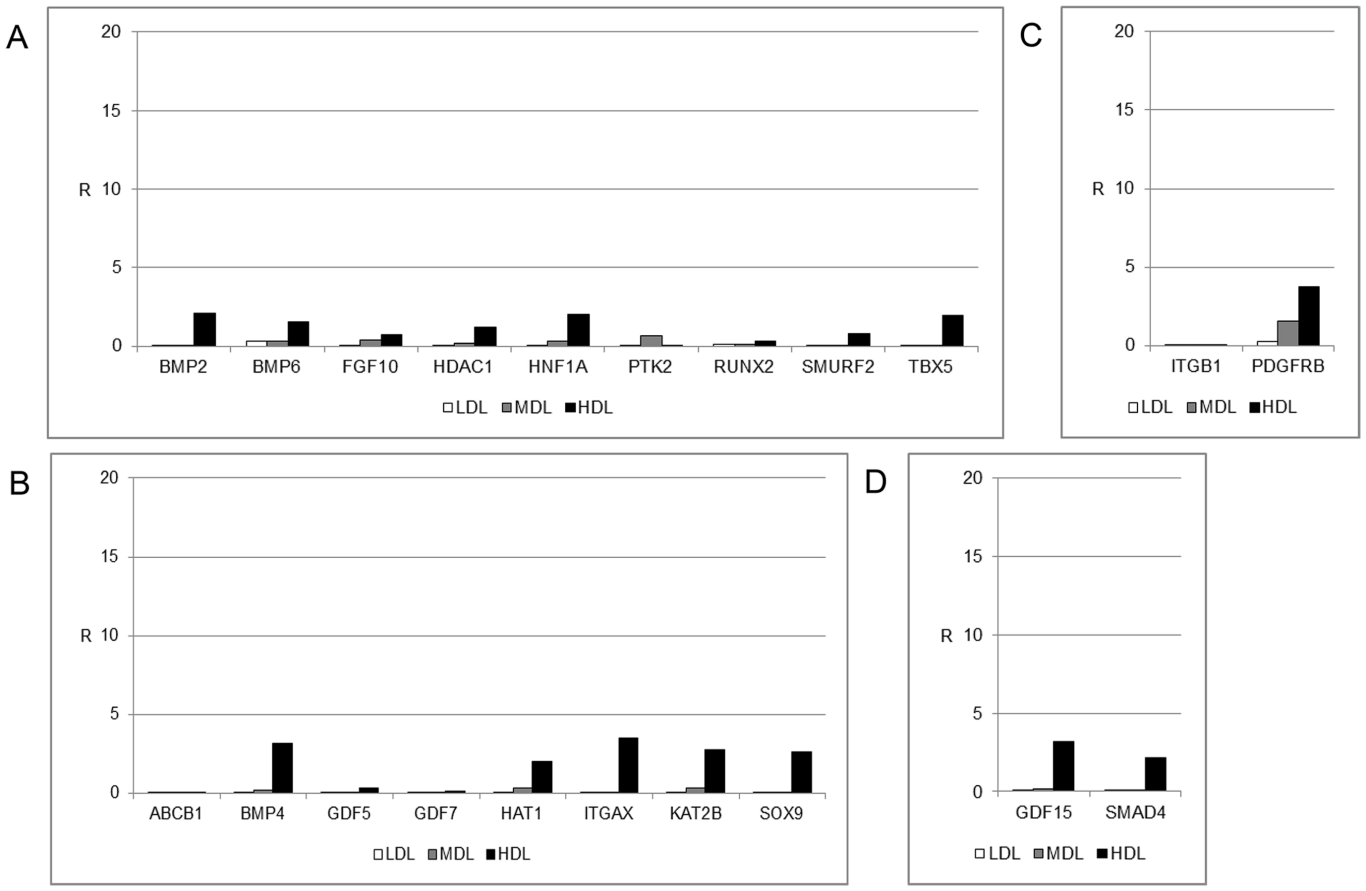
Gene expression profile of genes associated with mesenchymal stem cell markers. Gene expression profile of genes associated with mesenchymal stem cell markers in the LDL (white bars), MDL (grey bars), and HDL (black bars). The results are reported as ratios (R) with respect to the mRNA expression levels of non-centrifuged fat (not shown). (A) Osteogenesis, (B) chondrogenesis, (C) myogenesis, and (D) tenogenesis.

Finally, we focused our attention on the expression of specific mesenchymal stem markers, as shown in Figure 4. Several genes were expressed, including ANXA5, COL1A1, CTNNB1, EGF, FUT1, HGF, KITLG, MITF, MMP2, and VIM. High levels of vascular-inducing factors, such as VEGFA and VWF, have also been detected. No markers for commitment into osteogenic, chondrogenic, myogenic, or tenogenic lineages were present in the three layers after centrifugation (Figure 5).

**Figure 6:**
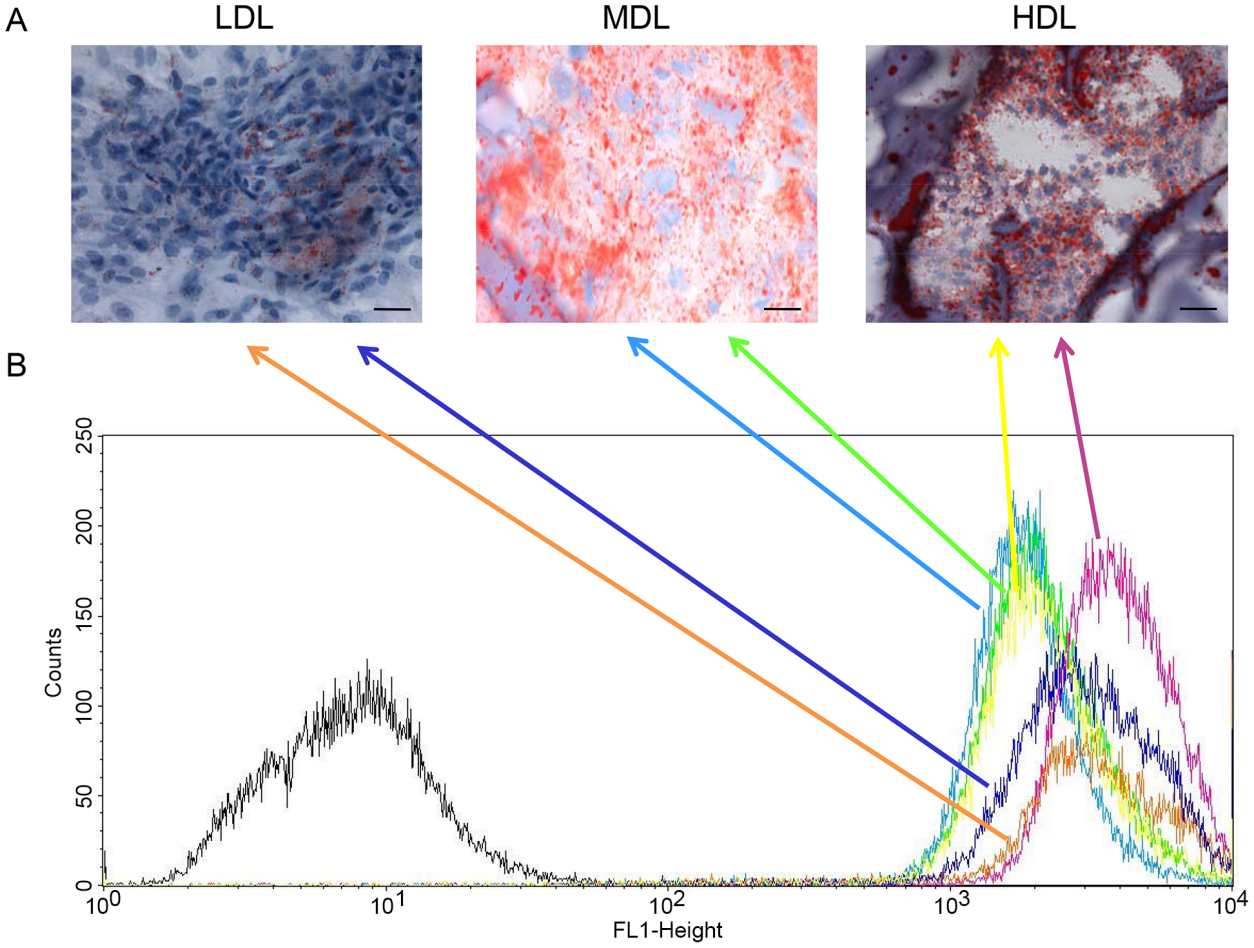
Evaluation of the lipid contents of the LDL, MDL, and HDL. (A) ORO staining (red drops in each panel) shows higher lipid contents in the MDL and in the HDL compared with that of the LDL (scale bar = 100 µm). (B) Semi-quantitative analysis of the lipid contents. The signals from the LDL are in blue and orange, signals from the MDL are in sky blue and green, and signals from the HDL are in yellow and purple.

Furthermore, we analyzed the adipogenic properties of each layer by measuring the lipid content. As shown in Figure 6A, each layer of fat displayed cells able to store lipids in their cytoplasm. Indeed, the cells from each layer were positively stained with ORO; however, higher quantities of lipid-containing cells were present in the MDL and in the HDL. Similar findings were obtained when we performed a semi-quantitative analysis (Figure 6B) of the staining inside the cytoplasm of cells from the different layers. The cells from the LDL stored less lipids than the cells from the bottom level.

**Figure 7:**
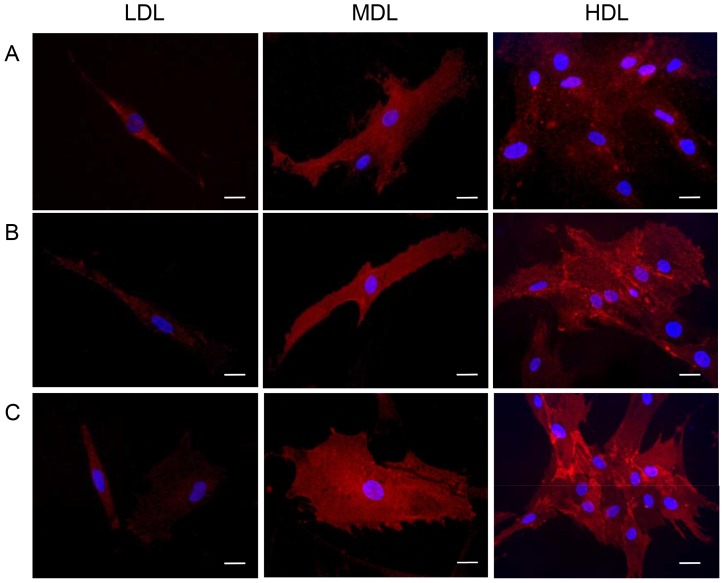
Immunostaining of stem cell markers present in the LDL, MDL, and HDL. Positive (red) cells for (A) CD73, (B) CD90, and (C) CD105 (scale bar = 10 µm).

We analyzed the presence of the specific stem cell markers CD73 (NT5E), CD90, and CD105 (ENG) using immunofluorescence. As shown in Figure 7, the positive cells for these markers were found in all layers. However, a greater number of positive cells was found in the HDL than in the LDL.

**Figure 8:**
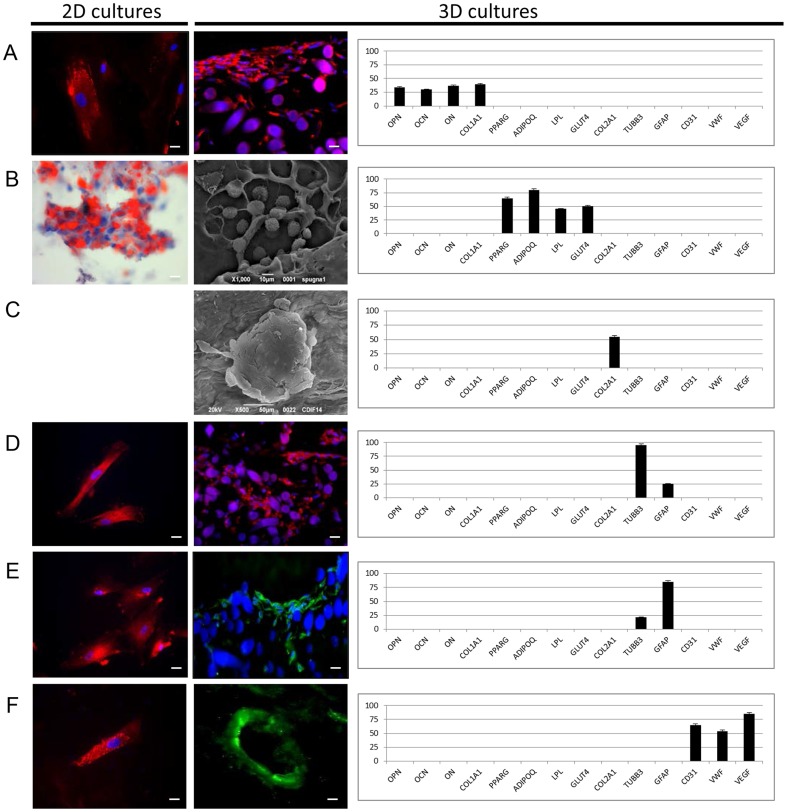
Commitment ability of stem cells present in the HDL. (A) Osteogenic commitment. Immunofluorescence confirms positive staining for osteonectin (red) in both 2D cultures (scale bar = 20 µm) and 3D cultures (scale bar = 50 µm). Real-time PCR detects the expression of osteopontin (OPN), osteonectin (ON), osteocalcin (OCN), and type I collagen (COL1A1), which are typical markers of osteogenic commitment. (B) Adipogenic commitment. ORO staining confirms lipid deposition inside the cytoplasm (red) in 2D cultures (scale bar = 50 µm). SEM analysis of 3D cultures reveals the typical adipogenic round shape of cells (scale bar = 10 µm). Genes related to the adipogenic phenotype, such as PPARG, LPL, GLUT4, and ADIPOQ, are expressed. (C) Chondrogenic commitment. In the 3D cultures, the cells are organized into their typical clusters, as shown by SEM analysis (scale bar = 50 µm), and only express type II collagen (COL2A1), as detected by real-time PCR. (D) Neuronal commitment. Immunofluorescence confirms positive staining for TUBB3 (red) in both 2D cultures (scale bar = 40 µm) and 3D cultures (scale bar = 100 µm). High expression of TUBB3 and low expression of GFAP are evident. (E) Glial commitment. Immunofluorescence confirms positive staining for GFAP in both 2D cultures (red, scale bar = 20 µm) and 3D cultures (green, scale bar = 100 µm), where significant GFAP mRNA expression is also detectable. (F) Endothelial commitment. Immunofluorescence confirms positive staining for VWF (red) in 2D cultures (scale bar = 20 µm) and for CD31 (green) in 3D cultures (scale bar = 100 µm). In the 3D scaffolds, the cells are also able to organize into micro-capillary vessels. Gene expression of endothelial markers, such as CD31 and VEGF, is clearly detectable.

The commitment ability of the stem cells present in the HDL was analyzed using in vitro experiments. Cells derived from the bottom level were seeded both as monolayers (2D culture) and on three-dimensional scaffolds (3D cultures) and cultured for up to 28 days in the presence of specific differentiation media (Figure 8). The commitment into osteogenic, adipogenic, chondrogenic, neuronal, glial, and endothelial lineages was detected using immunofluorescence and gene expression analyses. For the osteogenic commitment, the cells in monolayers and on non-woven HYA-based scaffolds were treated with an osteogenic inducer. As reported in Figure 8A, osteonectin expression was evaluated using immunofluorescence in both 2D and 3D cultures. Gene expression of 3D cultures was also used to better characterize this commitment. As reported in Figure 8A, genes for osteogenic commitment, such as osteopontin (OPN), osteonectin (ON), osteocalcin (OCN), and type I collagen (COL1A1), were expressed, whereas genes related to other lineages were not expressed. The results related to the adipogenic commitment are reported in Figure 8B. Morphological analyses performed with ORO staining of lipid drops indicated many positively stained cells. This result was also confirmed by SEM analyses of the 3D cultures (cells seeded onto HYA-based sponges), in which the typical adipogenic round shape was evident. Genes related to the adipogenic phenotype, such as peroxisome proliferator-activated receptor gamma (PPARG), lipoprotein lipase (LPL), solute carrier family 2 (facilitated glucose transporter), member 4 (GLUT4), and adiponectin (ADIPOQ), but not those genes related to other lineages, were expressed. For chondrogenic commitment, only 3D cultures were prepared because of the characteristics of this tissue. In this case, cells derived from the HDL were seeded onto a bovine-derived pericardial membrane and cultured with chondrogenic medium. After 28 days, the cells organized themselves into typical clusters and expressed only type II collagen (COL2A1) but no other markers (Figure 8C). Neuronal and glial commitment analyses were also performed on 3D HYA-based scaffolds, which were non-woven meshes. As reported in Figure 8D (for the neuronal commitment) and in Figure 8E (for the glial commitment), the cells expressed the neuronal marker beta III tubulin (TUBB3) and the glial marker glial fibrillary acidic protein (GFAP) at both protein and mRNA levels.

The endothelial properties of the stem cells present in the HDL were also analyzed (Figure 8F). Commitment was evaluated using positive endothelial markers, such as von Willebrand factor (VWF) and CD31, in both 2D and 3D cultures (on HYA non-woven meshes). In the 3D scaffolds, the cells organized into micro-capillary vessels. The gene expression of endothelial markers, such as CD31, VWF, and vascular endothelial growth factor (VEGF), was also present, without the expression of other cell markers.

## Discussion and Conclusions

Fat grafting has become popular as a stand-alone technique or as a part of a combined procedure for facial rejuvenation and for reconstructive craniofacial surgery [1]. Autologous tissue is completely biocompatible and is typically the safest choice for altering facial volume or contours. Fat grafts can be placed in such a manner that these grafts are long-lasting, completely integrated, and natural looking. The transfer technique is also known as structural fat grafting (SFG), or lipostructure, and requires the centrifugation of fat and its implantation into a specific site to be restored [29], [30]. Currently, no attention has been given to the stemness properties of the cells that are injected into the patient. In this study, we performed molecular analyses of the stemness properties of cells distributed in the fat following the centrifugation required by Coleman's procedure.

The distribution of stem cells across the three detected layers has been well defined. In the LDL, a few (but important) stem cell markers, such as ZFP42, were expressed (Figure 2). This finding indicates the presence of small multipotent stem cells in this layer (dimensions of 20–50 nm, data not shown). The MDL did not express stemness markers other than EGF. The stromal component was predominantly represented in the HDL, where high expression levels of mesenchymal stem cell markers (LIF, SOX2, WNT3A, CTNNB1, FUT4, EGF, KITLG, HGF, and VIM) were detected (Figure 3). Another notable finding is the high expression levels of endothelial markers (such as VEGFA) in this layer, indicating the high vasculogenic properties of the cells in this layer (dimension of the cells in this layer ranged from 50–100 nm, data not shown) (Figure 4). No specific markers for commitment into osteogenic, chondrogenic, myogenic, or tenogenic lineages have been detected (Figure 5); however, this absence is most likely the result of a lack of commitment conditions, such as specific medium, growth factors, or an appropriate scaffold. The expression of some inflammatory cytokines, such as IL1B and TNF, and of apoptotic molecules, including CASP3, has also been detected. Thus, the mechanical procedure of lipoaspiration may act as an inflammatory stimulus in the adipose tissue.

To better characterize the cell populations of each layer, we also analyzed the adipogenic properties of each layer by measuring the lipid contents (Figure 6) and by immunofluorescence analyses of cell-surface stem cell markers (Figure 7).

As shown in Figure 6, each layer contained cells able to store lipids in their cytoplasm, confirming the presence of mature adipocytes. We also analyzed the expression of the stem cell markers CD73, CD90, and CD105. As reported in Figure 7, we observed expression of these markers in all three layers; however, a greater number of positive cells were detected in the HDL than in the LDL.

Finally, the commitment ability of the stem cells present in the HDL was analyzed using in vitro experiments. Our results confirmed that, in presence of specific growth factors, cells differentiated into several lineages, including osteogenic, adipogenic, condrogenic, neuronal, glial, and endothelial lineages (Figure 8). The obtained results confirm that the distribution of stem cells into the three layers is well defined. This information is useful for surgeons who may apply the different layers in different situations. We speculate that the HDL contains a larger quantity of stem cells and a larger vascular potential compared with the other fractions; thus, this layer could be used for the regeneration of large defects. The medium layer, which has a larger quantity of differentiated cells, could be useful for increasing volume, and the layers enriched with multipotent cells are suitable for all uses.

In conclusion, we hypothesize that centrifugation creates a graded density of fat with varying characteristics that influence lipoaspirate persistence, properties, and quality.
